# The Therapeutic Value of Croton Chloral

**Published:** 1879-09

**Authors:** 


The Therapeutic Value of Croton Chloral.—In a very interesting paper read before the Ulster Medical Society, Dr. Riddell [*Dublin Medical Journal*, April, 1879] reports his experiences of the great therapeutical value of croton [butyl] chloral. He mentions a case of severe paroxysmal headache, ineffectually treated for many years by all the usual remedies of the Pharmacopœia, but cured by five grains of butyl-chloral twice daily and ten grains taken at night dissolved in spirits of wine and glycerine, with a little acid and syrup of orange to cover the flavor. The patient continues the five grain doses at night, and now enjoys better health than she has done for years. Since that case, Dr. Riddell says he has used it largely, sometimes relieving, till, by keeping an account of all his cases, it began to be clear which were most benefitted by the drug.

Since then the number of cases relieved [some permanently] has increased. These cures are: headache in females arising from mental distress; those cases of headache frequent at the menopause—in fact, all those called neuralgic, except a few arising from internal mischief, are benefitted, and in many instances cured. In that distressing species of neuralgia called tic douloureux he has found it in many cases acting like a charm. Of course, he does not include any arising from cranial or intracranial causes. He has tried it in neuralgia of the ovaries, but no good resulted. In insomnia, it is not so reliable as the hydrate: but in some cases, where the loss of or inability to sleep is accompanied by a weak or fatty heart, it is to be preferred, as it has no weakening effect on the central organ of the circulation.

In one case of delirium tremens, where the circulation was very feeble, the combination of croton chloral with digitalis had a wonderful effect, and it seemed as if the drugs could be given together in much smaller doses to produce the same results than singly. In this case he pushed it from ten to thirty grains every three hours, with drachm and two-drachm doses of the infusion of digitalis. In pain arising from caries of teeth, he has found it useless in most cases, and in all inferior to Richardson's "tinctura gelsemini," but in one case of a nervous young lady, by giving her two ten-grain doses, he was able to extract a tooth next to painlessly, to her great satisfaction.

It is in affections of those parts supplied by the fifth pair of nerves that it is of most use, but, to be of service, the drug must be given in far larger doses than prescribed in the Pharmacopœia for adults—five grains three or four times daily, gradually increasing if required. If stimulants be wanted, dissolve it in rectified spirit; if not, dissolve it in glycerine. In all cases complicated with hæmorrhoids, give glycerine. If anæmia exist, combine it with iron, or, what he believes better, arsenic; then gradually lessen the chloral. In all cases he has found it better to give it in solution than in powder or pill. Dr. Riddell mentions also severe pain with photophobia and blepharospasm after injury, in which atropia failed, but ten grains of butyl-chloral, repeated in an hour, gave complete relief; and a case of acute painful facial carbuncle, in which the effect of ten-grain doses every three hours was "simply marvellous," the disease going through its frequent stages almost without the patient knowing anything of the matter from the sense of feeling.—*British Medical Journal.*

